# From cholera to corals: Viruses as drivers of virulence in a major coral bacterial pathogen

**DOI:** 10.1038/srep17889

**Published:** 2015-12-08

**Authors:** Karen D. Weynberg, Christian R. Voolstra, Matthew J. Neave, Patrick Buerger, Madeleine J. H. van Oppen

**Affiliations:** 1 Australian Institute of Marine Science, PMB #3, Townsville 4810, Queensland, Australia; 2 Red Sea Research Center, Division of Biological and Environmental Science and Engineering, King Abdullah University of Science and Technology (KAUST), Thuwal 23955-6900, Jeddah, Saudi Arabia; 3 School of Marine and Tropical Biology, James Cook University, Townsville 4811, Queensland, Australia; 4 AIMS@JCU, Townsville, Queensland 4814, Australia; 5 School of BioSciences, The University of Melbourne, Parkville, Melbourne, 3010, Victoria, Australia

## Abstract

Disease is an increasing threat to reef-building corals. One of the few identified pathogens of coral disease is the bacterium *Vibrio coralliilyticus*. In *Vibrio cholerae*, infection by a bacterial virus (bacteriophage) results in the conversion of non-pathogenic strains to pathogenic strains and this can lead to cholera pandemics. Pathogenicity islands encoded in the *V. cholerae* genome play an important role in pathogenesis. Here we analyse five whole genome sequences of *V. coralliilyticus* to examine whether virulence is similarly driven by horizontally acquired elements. We demonstrate that bacteriophage genomes encoding toxin genes with homology to those found in pathogenic *V. cholerae* are integrated in *V. coralliilyticus* genomes. Virulence factors located on chromosomal pathogenicity islands also exist in some strains of *V. coralliilyticus*. The presence of these genetic signatures indicates virulence in *V. coralliilyticus* is driven by prophages and other horizontally acquired elements. Screening for pathogens of coral disease should target conserved regions in these elements.

Species of the gammaproteobacterium *Vibrio* are well-known for their roles as pathogens in both terrestrial and aquatic environments^1^. However, not all species and strains of *Vibrio* are pathogenic^2^,^3^. For instance, *Vibrio cholerae*, a bacterium that causes the acute diarrhoeal disease cholera^4^,^5^,^6^ requires the acquisition of key virulence factors to become toxigenic. The two major virulence factors of *V. cholerae*, TCP (toxin co-regulated pilus) and an exotoxin called cholera toxin (CTX), are both introduced via horizontal gene transfer (HGT) and are encoded by a pathogenicity island^7^ and the genome of a single-stranded DNA lysogenic filamentous bacteriophage (or prophage) called CTXphi (CTXφ)^6^, respectively. The TCP receptor is a prophage receptor facilitating introduction of CTXφ prophage DNA into the cell^6^. In *V. cholerae*, CTXφ prophage transmits toxin genes among the bacteria that express the TCP prophage receptor. This process of inducing virulence in an otherwise harmless bacterium through prophage infection and integration of the prophage genome into the bacterial host genome is called lysogenic conversion^8^.

The health of coral reefs is declining on a global scale^9^,^10^. One of the causes of this deterioration is coral disease, the incidence and severity of which has increased worldwide over recent decades^11^,^12^,^13^. A number of coral disease studies have reported *Vibrio* species, *V. coralliilyticus* in particular, as the pathogen responsible for some instances of white syndrome (WS) disease^14^,^15^, as well as coral bleaching^16^,^17^. Coral bleaching is the loss of the algal endosymbiont, *Symbiodinium,* and/or its photosynthetic pigments from the coral tissue resulting in paling of the coral tissues. If these coral algal endosymbionts are not regained following bleaching, coral mortality is inevitable^18^. Bleaching tends to occur more gradually in comparison to the acute disease of WS that results in rapid and irreversible loss of tissue biomass (between 1 to 100 cm^2^ day^−1^)^19^. Tissue loss can result in white spots, bands or patches of exposed skeleton with a distinct lesion boundary between exposed skeleton and visibly healthy tissue^17^,^19^.

Pathogenicity of the coral-associated *V. coralliilyticus* in coral disease and bleaching has been attributed to increases in sea surface water temperatures^14^,^20^, and the immunocompetence of the coral host^21^. However, the underlying mechanisms are not understood, and a possible role for prophages in driving virulence of coral pathogens, as is the case for *V. cholerae*, has not yet been considered. Inconsistent findings on the role of *Vibrio* in coral disease/bleaching events have been reported in the literature^16^,^22^,^23^. In the case of the coral pathogen *V. coralliilyticus*, identified as the causative pathogen of WS in the coral *Pocillopora damicornis*^14^,^15^, it was initially observed that *V. coralliilyticus* encodes for a zinc-metalloprotease that causes cellular damage in coral tissue^17^,^24^. However, a recent study did not detect differences in pathogenicity of a *V. coralliilyticus* strain (P1) between a wild-type and zinc-metalloprotease-deficient mutant in infection experiments^25^, indicating that factors other than zinc-metalloproteases (e.g. hemolysin activity; other proteases) produced by *V. coralliilyticus* strains may also be at play and contribute to virulence. Moreover, the possible role of a prophage as an alternative driver of virulence in *V. coralliilyticus* in these studies has not been addressed.

The aim of this study was to assess whether a prophage could potentially drive virulence of *V. coralliilyticus* by examining *in silico* five publically available *V. coralliilyticus* whole genome sequences^20^,^25^,^26^,^27^,^28^. Our focus was on the presence of prophage signatures and virulence factors in these genomes, to identify lysogenic conversion by a prophage. Our analysis demonstrates the presence of lysogenic prophages in *V. coralliilyticus* genomes that share similarities to prophages integrated in pathogenic *V. cholerae* strain genomes and supports our hypothesis.

## Results

Recent studies have reported five whole genome sequences of *V. coralliilyticus* associated with corals found in different locations worldwide (Table 1). The first strain of *V. coralliilyticus* to be sequenced was isolated from diseased and bleached corals off the coast of Zanzibar (*V. coralliilyticus* ATCC-BAA-450^14^,^20^ referred to here as BAA450). Subsequently, whole genome sequences were published for a strain isolated from a Great Barrier Reef coral (*V. coralliilyticus* P1^15^,^25^), and two strains isolated from corals located in Hawai’i (*V. coralliilyticus* OCN008^28^ and *V. coralliilyticus* OCN014^27^ ). Most recently, a fifth complete genome was sequenced from *V. coralliilyticus* isolated from oysters at a shellfish hatchery (*V. coralliilyticus* RE98^26^). We interrogated each genome for signatures of or complete prophage genomes.

### Prophages in *V. coralliilyticus* genomes

**Figure 1:**
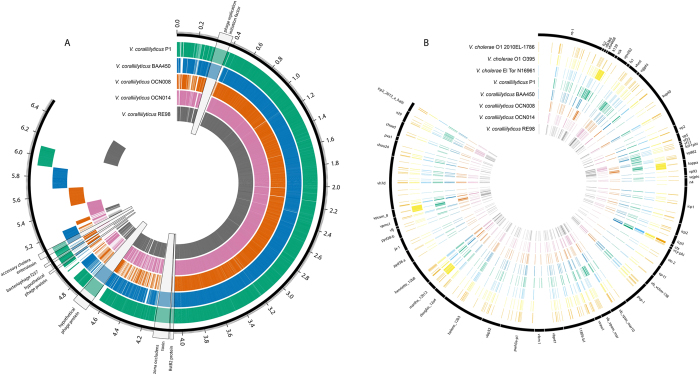
(**a**) Pangenome of the five *Vibrio coralliilyticus* strains. OrthoMCL v2.0.9 was used to generate clusters of similar proteins, which are indicated by overlapping regions. The tick marks indicate size of the pangenome in millions of base pairs (Mbps) and key prophage signatures are highlighted and magnified 100x. (**b**) Clustering of *Vibrio coralliilyticus* proteins and *Vibrio cholerae* proteins with known *Vibrio* phage proteins. OrthoMCL v2.0.9 was used to detect similarity between proteins in the *V. coralliilyticus* genomes and phage genomes, which are indicated by the overlapping tracks. To identify related protein clusters a minimum similarity of 50% and an e-value of <10^−5^ was used. Overlapping lines denote the presence of a similar protein in each of the genomes. The genomes each have a unique colour and this is not related to similarity. The outer-most numbers in plot A only give a guide to the total size of the pan-genome; these do not represent locations within genomes. The orthoMCL program looks only for similar proteins with no regard to their location or synteny within different genomes and therefore, this plot should only be used to determine the presence or absence of particular proteins of interest.

**Figure 2:**
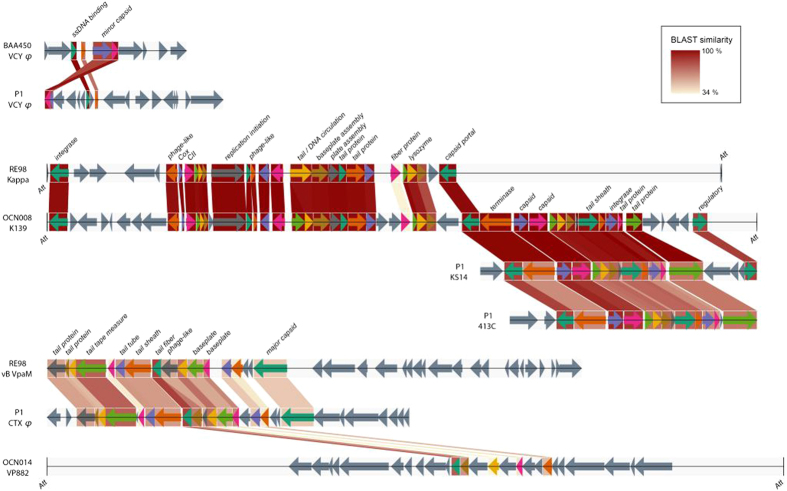
Genome organisation of prophages found in the genomes of five *Vibrio coralliilyticus* strains isolated from corals and oysters. BLAST values were determined using amino acid sequences with BLASTP. Linear ORF maps of the prophages were aligned based on their modular structures. ORFs or genes are represented by arrows oriented in the direction of transcription. Coloured arrows are annotated to indicate predicted function. Grey arrows represent unknown ORFs. The *att* (phage attachment sites), where identified, are indicated.

**Figure 3:**
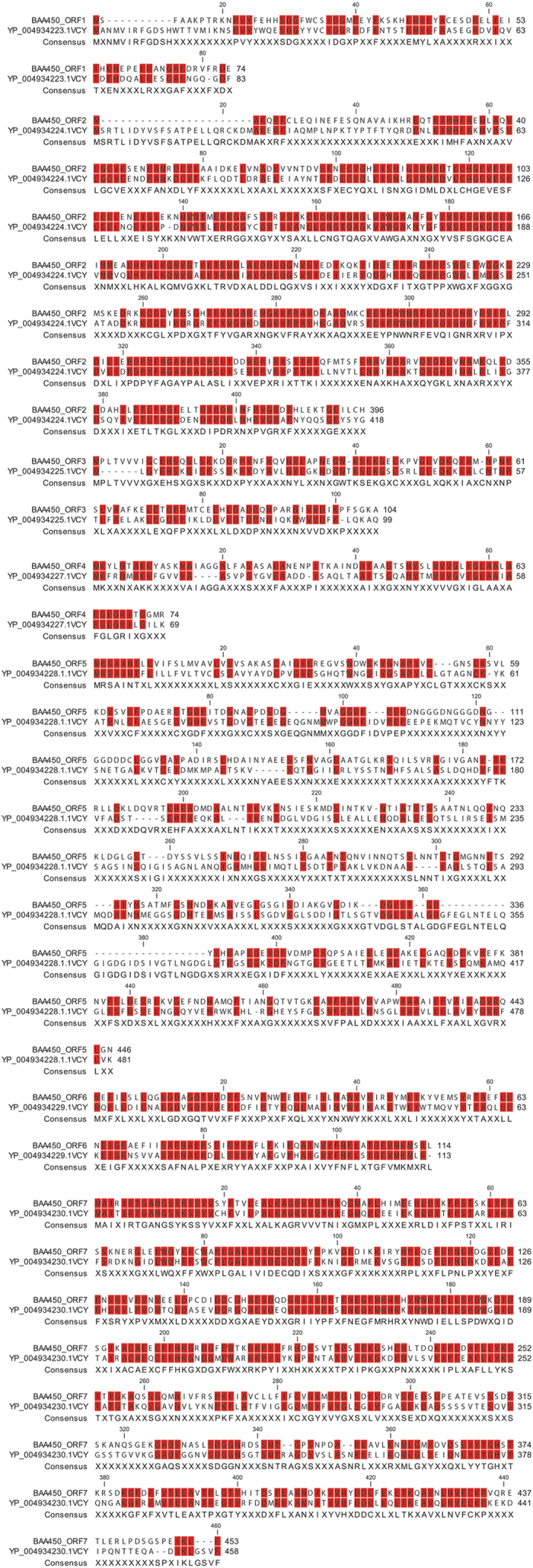
Amino-acid alignment of 7 of 9 ORFs encoded by the BAA450 prophage with 7 of 11 ORFs in the *Vibrio* phage VCY-phi (Accession No. NC_016162.1). Conserved residues are shaded in red. Dashes indicate gaps.

Our *in silico* analysis revealed the BAA450 genome contains an intact prophage genome that shares homology to the core region of the CTXφ genome (Figs 1 and 2) and encodes a number of genes that share close identity to those encoded by a filamentous prophage, VCYφ, isolated from an environmental *V. cholerae* population^29^ (Tables 2 and 3; Figs 2 and 3). The VCYφ prophage genome shares sequence similarity to the CTXφ prophage^29^, and like CTXφ, is a novel virus that has evolved through HGT events and recombination. A prophage genome encoding a number of genes homologous to those encoded by VCYφ was also detected in the *V. coralliilyticus* P1 strain but is absent from the OCN008 and OCN014 strains from Hawai’i and the RE98 genome (Tables 2 and 3; Figs 1 and 2).

For the morphogenesis of the CTXφ prophage, three genes known as *zot* (zonal occludens toxin), *ace* (accessory cholera enterotoxin) and *cep* (core-encoded cepin) are required^30^. Both the *zot* and *ace* genes are identified as minor coat proteins of the CTXφ prophage but also exhibit toxic activity by encoding for toxins that play a direct role in disrupting the intestines of humans with cholera disease^30^. A *rst*B gene (Figs 1a and 2) encodes a DNA-binding protein essential for prophage formation and is identified in the CTXφ genome as playing a role in the site-specific integration of the prophage into the host chromosome^31^. Homologues of these genes are encoded by the genome of the *Vibrio* VCYφ –like prophage found in BAA450 and P1 (Table 3; Figs 1, 2, 3; Supplemental Figure 1). Identity scores for specific genes for these prophages in BAA450 and P1 are shown in Table 3. Sequence alignment of key genes in the BAA450 prophage genome with similar genes encoded by VCYφ genome is shown in Fig. 3. Genes in the BAA450 prophage that share close similarity with those in the VCYφ genome include a DNA replication initiation protein (identity = 66%; score = 559), a gene encoding a ssDNA-binding protein, similar to the *rst*B gene (identity = 53%; score = 103), a minor capsid protein gene similar to *ace* (identity = 29%; score = 158) and a gene encoding a Zot-like protein (identity = 61%; score = 578) (Table 3).

An intact prophage was detected in the OCN014 genome that shares similarity to a plasmid-like prophage VP882^32^ found in *V. parahaemolyticus*, a pathogen commonly associated with shellfish (Table 2; Figs 1b and 2). This prophage genome shares similarity to a small number of genes in the *Pseudomonas* phage CTXφ-like genome found in P1 (Fig. 2). PHAST^33^ analysis of the OCN008 genome detected one intact prophage that shares similarity with a *V. cholerae* prophage K139^34^ and also shares homology with the incomplete Kappa prophage located in the RE98 genome (Table 2; Figs 1b and 2). The phage K139 has been previously identified as associating with pathogenic *V. cholerae* strains but it is still unclear whether or how this prophage may increase pathogenic host fitness^35^. Strain RE98 has been identified as a particularly virulent pathogen responsible for high mortality rates in oyster shellfish hatcheries in the west coast of the US^36^. A second intact vB VpaM-like prophage was detected in the RE98 genome that shares homology to the *Pseudomonas* CTXφ-like prophage in P1 (Table 2: Figs 1b and 2). A heat map analysis indicates similarity between the prophage genomes identified in this study (see Supplementary Fig. S1 online).

### Pathogenicity islands in the *V. coralliilyticus* genome

The synergistic effect of a number of genes, including toxicity genes introduced by CTXφ, and a series of genes clustered in pathogenicity islands on the bacterial genome result in the evolution of *V. cholerae*^7^ to harmful epidemic and pandemic-causing strains. The pathogenicity islands (namely Vibrio Pathogenicity Island I and II, VPI-I/II) consist of virulence genes, a transposase gene, specific (att-like) attachment sites flanking each end of the island, and an integrase with homology to a prophage integrase gene^7^. The TCP gene cluster induces virulent *Vibrios* to secrete the exotoxin cholera toxin (CTX) and is encoded on a pathogenicity island within the *Vibrio* chromosome^7^. These genes are present in BAA450 and P1, suggesting these elements may influence the virulence of BAA450 and P1. Both BAA450 and P1 genomes carry a Coralliilyticus Pathogenicity Island-I (CPI-I) that is located at a chromosomal site similar to the *dif*-like region that accommodates an insertion site for the prophages CTXφ and f237 of *V. cholerae*^6^ and *V. parahaemolyticus*^37^, respectively. The TCP is encoded for in CPI-1 of BAA450 and P1. While BAA450 has a similar genome arrangement to *V. cholerae* with the presence of a second pathogenicity island (CPI-II)^20^, the P1 genome does not contain a second pathogenicity island CPI-II. It is noteworthy that the CPI-II in BAA450 bears similarity to a second pathogenicity island found in the genome of the seventh pandemic strain of *V. cholerae*^7^. Further, of the five genomes analysed here, the BAA450, P1, OCN014 and RE98 genomes encode for a RTX (repeats in toxin) protein that is a harmful pore-forming toxin required for human host colonisation in incidences of infection by *V. cholerae*^30^. The RTX gene cluster in *V. cholerae* is physically located close to the CTXφ genome^38^. A significant upregulation of the RTX protein and a number of proteins located on the same pathogenicity island as the RTX protein occurs in BAA450 at increased temperatures of 27 °C and above, contributing to increased virulence at higher temperatures^20^.

## Discussion

Prophage infections can transform ordinary bacteria to virulent pathogens, shown in a wide range of species including *Vibrio cholerae*^6^, *Staphylococcus aureus, Clostridium botulinum, Corynebacterium diphtheriae, Streptococcus pyrogenes* and *Escherichia coli*^8^,^39^. A landmark discovery in bacteriophage research revealed the role of lysogenic prophages in the conversion of the bacterium *Vibrio cholerae* from a non-pathogenic to a virulent strain, resulting in often devastating cholera outbreaks among human populations^4^,^5^,^6^. Our analysis shows that the same scenario may exist for the coral pathogen, *Vibrio coralliilyticus,* and future studies should rigorously address this previously overlooked possibility.

BAA450 was reported over a decade ago as causing bleaching and tissue necrosis in the coral species *Pocillopora damicornis*^14^,^17^. This strain was seen to induce bleaching at temperatures between 24–26 °C, but not at lower temperatures. An increase in protease activity resulting in the release of high amounts of toxic extracellular proteases was observed at temperatures between 27–29 °C, which resulted in mortality of the coral^17^. The presence of an intact prophage genome in the BAA450 genome and an incomplete prophage in P1 that share similarities to the genome of CTXφ prophage, responsible for toxigenic conversion of *V. cholerae* strains, suggests that similar prophage-driven virulence mechanisms could operate in both the human and coral system. Extracellular zinc-metalloproteases expressed by *V. coralliilyticus* strain P1 were previously reported to be the driver of tissue damage in WS infections^17^,^24^ but these enzymes were not the only drivers as demonstrated by subsequent mutant infection experiments^25^. These studies did not include an assessment of the presence of CTXφ prophage-derived toxins, such as ZOT and ACE proteins. We therefore encourage future experiments towards assessing the expression of these virulence factors in infections by *V. coralliilyticus* strains.

The OCN008 strain has been indicated as an aetiological agent in white syndrome of the coral species *Montipora capitata*^28^. Unlike strains BAA450 and P1, the pathogenesis of OCN008 is reported as being temperature-independent^28^ with only a relatively slower onset of infection observed at lower temperatures, resulting in tissue loss without bleaching. Previous comparative analysis of the genome sequences of BAA450, P1 and OCN008 reported approximately 84% similarity between the genomes of OCN008 and BAA450 and 89% similarity between the OCN008 and P1 genomes^28^. Genes upregulated at least 4-fold in BAA450 by a rise in temperature shared 97–100% sequence identity with genes in OCN008^28^. Why does infection by OCN008 appear to be independent of increasing temperature? The intact prophage genome detected in the OCN008 genome shares similarity to the *Vibrio* prophage K139 that is also present in the *V. cholerae* O1 O395 genome (Fig. 2). It is possible that the presence of this intact prophage in the OCN008 genome and the absence of a CTXφ-like prophage genome results in a different disease profile compared to those of the BAA450 and P1 temperature-dependent infections. OCN014 has been isolated from corals exhibiting symptoms of WS^27^. Further investigations are needed to link the presence of OCN014 to coral disease and we encourage the inclusion of virus characterisation in such experimental work.

*V. cholerae* is autochthonous to coastal and estuarine waters and rising seawater temperatures have been indicated as a major influence on *V. cholerae* pandemics^40^. Does the presence of a prophage similar to the CTXφ prophage in the BAA450 and P1 genomes explain these strain’s increased virulence with rising temperature^14^,^17^,^41^,^42^? It has been predicted, based on time-series studies, that as global temperatures rise, cholera outbreaks will increase^40^,^43^,^44^. Comparably, researchers have indicated the role of seasonal dynamics, microbial interactions and environmental factors driving the occurrence of disease caused by *V. coralliilyticus* and these are predicted to increase with ongoing environmental change^16^,^41^,^45^. For instance, a recent study reported *V. coralliilyticus* populations in *P. damicornis* increased by as much as four times with elevated seawater temperatures^46^. We postulate that as temperatures increase the BAA450 and P1 prophages will undergo increased active replication along with host genome replication, and hence, their associated virulence factors (e.g. ZOT and ACE proteins, as well as TCP and RTX) will increase. With a changing climate, sea surface temperatures have steadily risen accompanied by expanding coral pathogen ranges^47^.

There have been greater than 30 coral diseases described^41^,^48^ but only a few diseases have been attributed to causative bacterial pathogens, namely *Thalassotalea loyana* (formerly *Thalassomonas loyana*) in white plague disease^49^, *Vibrio shiloi* in bleaching of the coral *Oculina patagonica*^16^,^50^ and *V. coralliilyticus* in coral bleaching and white syndrome disease^14^,^15^,^17^. Coral disease profiling is complex and suspected pathogens of disease have been identified in apparently healthy corals^41^,^51^. The aetiology and pathogenicity of *V. coralliilyticus* has generated much interest, but very little is understood about the dynamics of infection. The parallels between the *Vibrio cholerae* paradigm and an equivalent scenario in corals is highly relevant and comparable, as disease in both human and coral populations are influenced by environmental factors that are being altered through anthropogenic influences and changes in climatic conditions.

We encourage further experimental studies into the role of viruses in *Vibrio* infections in corals. Previous studies have described the presence of viruses associated with coral diseases^52^,^53^,^54^ but none have investigated the role of prophages and lysogenic conversion. As all *V. coralliilyticus* genomes sequenced to date have been isolated from corals with disease/bleaching symptoms, at present we cannot make comparisons and form conclusions on the presence of these prophages in genomes of *V. coralliilyticus* isolated from disease-free samples. We anticipate future sequencing efforts to include genomes of *V. coralliilyticus* from healthy corals, or other such sources, to enable an in-depth comparison. In this present study, we indicate possible signatures to target in *V*. *coralliilyticus* to screen for prophages. We envisage our *in silico* analysis to be a foundation and starting point for future experimental research on the role of lysogenic conversion in pathogenicity of *Vibrio* and the environmental conditions that potentially trigger virulence. Coral disease is not caused by a single factor but is the result of a complex interplay among biotic and abiotic factors and prophages are a potential central driver of virulence.

## Methods

For interrogative and comparative analysis, whole genome sequences of five published *V. coralliilyticus* genomes were downloaded from the NCBI database, March 2015. The *V. coralliilyticus* strain names and their accession numbers are as follows and summarised in Table 1: *V. coralliilyticus* ATCC-BAA-450^14^,^20^ accession number ACZN00000000.1; *V. coralliilyticus* P1^15^,^25^ accession number AEQS00000000.1; *V. coralliilyticus* OCN008^28^ accession number AVOO00000000.1; *V. coralliilyticus* OCN014^27^ accession numbers CP009264 and CP009265; and *V. coralliilyticus* RE98^26^ accession numbers CP009617 and CP009618 both OCN014 and RE98 genomes have been deposited under BioProject PRJNA224116. The five *V. coralliilyticus* genome sequences were curated and analysed using Ortho MCL v2.0.9^55^, an analysis tool that identifies orthologous groups for use in comparative genomics and evolutionary processes. Three *V. cholerae* representative reference genomes were downloaded from NCBI: *V. cholerae* O1 2010EL-1786^56^ GenBank accession number CP003069 and CP003070; *V. cholerae* O1 O395^57^ GenBank accession number CP000626 and CP000627; *V. cholerae* El Tor N16961^58^ GenBank accession number AE003852 and AE003853 and were also included in an OrthoMCL analysis. Comparative genomic analysis was visualised using the software tool Circos^59^ version 0.66. To detect integrated prophage genomes a PHAST (Prophage Search Tool, updated 01/05/2015)^33^ analysis was conducted on the five *V. coralliilyticus* genomes.

## Additional Information

**How to cite this article**: Weynberg, K. D. *et al.* From cholera to corals: Viruses as drivers of virulence in a major coral bacterial pathogen. *Sci. Rep.* **5**, 17889; doi: 10.1038/srep17889 (2015).

## Supplementary Material

Supplementary Information

## Figures and Tables

**Table 1:** Description of the five whole genome sequences of *Vibrio coralliillyticus* (four isolated from corals and one from oysters) analysed in this study and implicated in diseases of corals and oysters.

*Vibrio coralliilyticus* strain	Geographical origin of isolation	Species strain isolated from	Genome Accession No.	Genome size (Mb)	Disease state	Reference
BAA450	Zanzibar, Tanzania (ATCC, USA)	*Pocillopora damicornis*	ACZN00000000.1	5.68063	Bleaching/White syndrome	Kimes *et al.*, 2012^20^
P1	Nelly Bay, Magnetic Isld, Great Barrier Reef, Australia	*Montipora aequituberculata*	AEQS00000000.1	5.51326	White syndrome	Sussman *et al.*, 2008^15^; Santos *et al.*, 2011^25^
OCN008	Kaneohe Bay, Hawaii, USA	*Porites compressa*	AVOO00000000.1	5.5349	White syndrome	Ushijima *et al.*, 2014^28^
OCN014	Palmyra Atoll	*Acropora cytherea*	CP009264; CP009265	5.732794	White syndrome	Ushijima *et al.*, 2014^27^
RE98	Oregon, USA	Pacific oyster *Crassostrea gigas*	CP009617; CP009618	6.03782	Shellfish pathogen	Richards *et al.*, 2014^26^

**Table 2:** Details of prophages identified in five publically available *Vibrio corallilyticus* (VC) genomes.

VC strain	No. of prophage identified	Prophage genome length (kb)	Prophages (closest similarity) identified in VC genome	Score	Number of CDS	Region position	GC%
BAA450	1	7.8	Intact VCYφ phage (NC_016162)	95	9	1436462–1444336	43.64
P1	4	15.3	Incomplete Burkholderia- like phage KS14 (NC_015273)	60	18	511097–526448	40.42
		20	Intact PseudomonasφCTX phage (NC_003278)	140	29	1856473–1876563	45.13
		13.7	Incomplete Yersinia phage L-413C (NC_004745)	60	16	2505529–2519246	45.16
		9.9	Incomplete VCYφ phage (NC_016162)	20	16	5482028–5491938	46.0
OCN008	1	39.3	Intact Vibrio phage K139 (NC_003313)	130	52	1044704–1084092	44.12
OCN014	1	40.8	Intact Vibrio phage VP882 (NC_009016)	140	22	1698322–1739200	44.66
RE98	2	37.3	Incomplete Vibrio Kappa phage (NC_003313)	90	28	653256–690638	44.12
		13.3	Intact Vibrio vB VpaM phage (NC_019722)	130	17	934714–948029	46.28

**Table 3:** Similarity values for ORFs in *V. corallilyticus* (VC) strains BAA450 and P1 genomes with closest matches to VCYφ phage genes.

VC strain CDS position	VCYφ Accession No.	Gene identified	Identity (%)	E-value	Score	Query coverage (%)
BAA450_1	YP_004934223.1	Hypothetical protein	43	1e-13	66	81
BAA450_2	YP_004934224.1	DNA replication initiation protein	66	0	559	98
BAA450_3	YP_004934225.1	ssDNA-binding protein (RstB)	53	9e-28	103	88
BAA450_4	YP_004934227.1	Hypothetical protein	47	2e-10	57	100
BAA450_5	YP_004934228.1	Minor capsid protein	29	5e-41	158	99
BAA450_6	YP_004934229.1	Hypothetical protein	58	3e-41	139	99
BAA450_7	YP_004934230.1	ZOT-like protein	61	0	578	96
P1_1	YP_004934229.1	Hypothetical protein	47	4e-17	75.5	100
P1_2	YP_004934225.1	Minor capsid protein	43	4e-06	48	85
P1_7	YP_004934225.1	ssDNA-binding protein (RstB)	52	4e-09	53.5	89
P1_8	YP_004934226.1	Hypothetical protein	54	2e-17	75.5	100
P1_9	YP_004934227.1	Hypothetical protein	52	5e-07	47	94
