# Supplementary material for: From cholera to corals: Viruses as drivers of virulence in a major coral bacterial pathogen
Source: Sci Rep. 2015 Dec 8;5:17889. doi: 10.1038/srep17889 (PMC4672265; doi:10.1038/srep17889)
Supplement: Supplementary Information [file srep17889-s1.pdf]

From cholera to corals: Viruses as drivers of virulence in a major coral bacterial pathogen

Karen D. Weynberg[a*], Christian R. Voolstra[b], Matthew J. Neave[b], Patrick Buerger[a,c,d], Madeleine J. H. van Oppen[a,e]

[a]Australian Institute of Marine Science, PMB #3, Townsville 4810, Queensland, Australia
[b]Red Sea Research Center, Division of Biological and Environmental Science and Engineering, King Abdullah University of Science and Technology (KAUST), Thuwal 23955-6900, Jeddah, Saudi Arabia
[c]School of Marine and Tropical Biology, James Cook University, Townsville 4811, Queensland, Australia
[d]AIMS@JCU, Townsville, Queensland 4814, Australia
[e]School of BioSciences, The University of Melbourne, Parkville, Melbourne, 3010, Victoria, Australia
*Corresponding author

Supplementary material

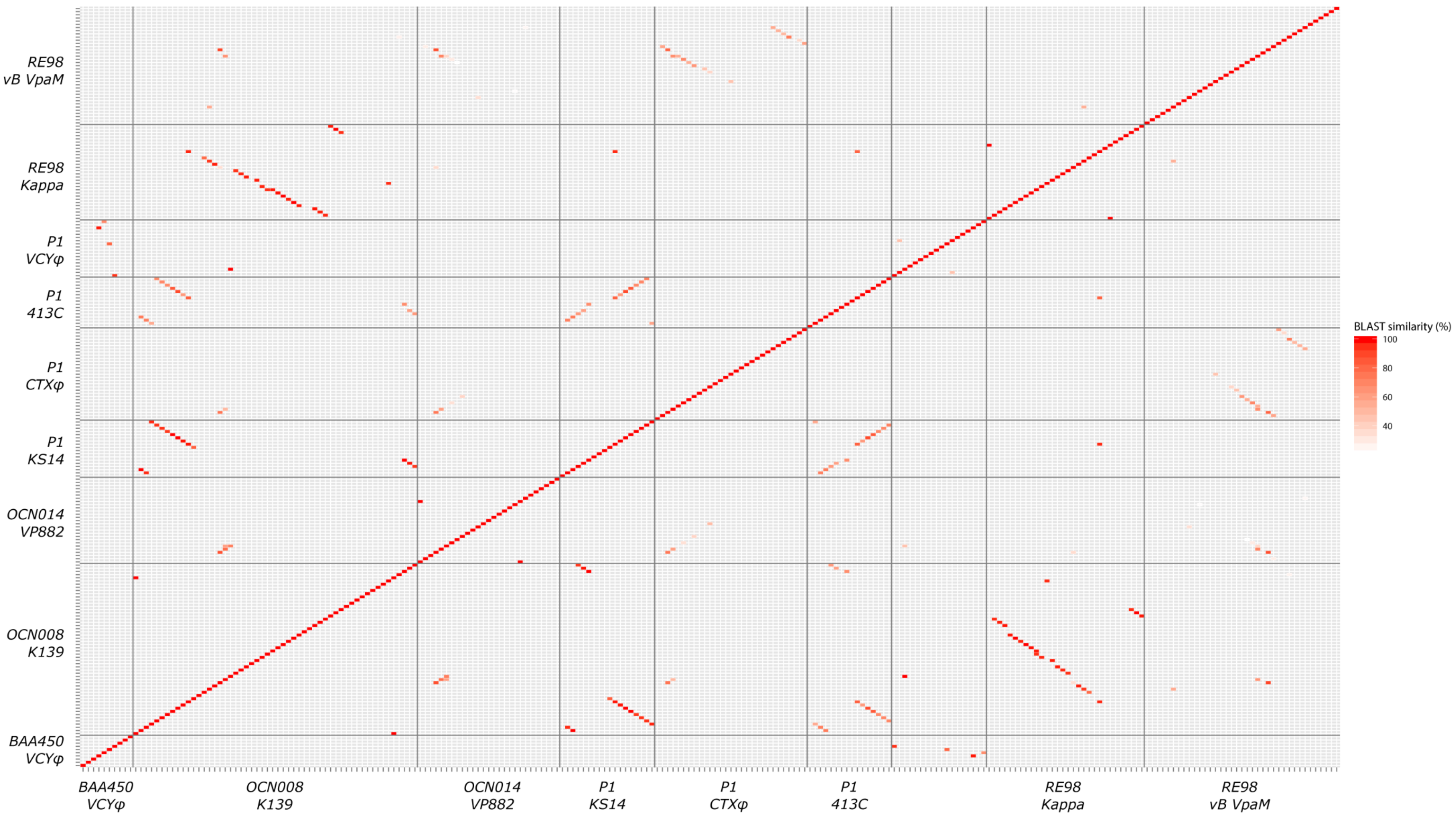

Supplementary Figure S1. Heat map analysis of all prophage genomes detected in five publically available Vibrio corallilyticus genomes.
